# 27042 Pressure-pain thresholds at baseline and in response to isometric exercise in Achilles tendinopathy

**DOI:** 10.1017/cts.2021.417

**Published:** 2021-03-30

**Authors:** Lauren Sara, Karis Yang, Meggie Rose Hart, Marie Hoeger Bement, Sandra Hunter

**Affiliations:** Marquette University

## Abstract

ABSTRACT IMPACT: Baseline presentation in AT (higher upper trapezius PPT with no difference at calf or tendon) may suggest a mechanism for persistent symptoms: with more advantageous central pain processing and no tradeoff peripherally, they may choose to continue their usual activities without regard for further damage to the affected tendon. OBJECTIVES/GOALS: Exercise-induced hypoalgesia, a reduction in pain with exercise, is often observed in healthy populations but is not well established in Achilles tendinopathy (AT). The aim was to compare pressure-pain threshold (PPT) at baseline and after fatiguing isometric exercise in AT and healthy controls. METHODS/STUDY POPULATION: 21 participants were recruited for the study: 7 AT (26.5 ±8.8 yrs), 14 control (22.1 ±3.2 yrs). After a familiarization session, participants completed an experimental session that involved performance of intermittent maximal voluntary isometric contractions (MVICs) (2s:2s duty cycle) in a Biodex3 dynamometer (Biodex Medical, Shirley, NY) for 4 minutes. PPT was measured at the medial gastrocnemius (calf), Achilles tendon, and upper trapezius at baseline and immediately following the fatiguing isometric task using a Somedic Algometer (Somedic AB, Sweden). Data are expressed as Mean(SD). Change in PPT is expressed as a percentage of baseline PPT. Units for PPT are kPa. A priori alpha was set to 0.05. RESULTS/ANTICIPATED RESULTS: There was no change in tendon or calf PPT following isometric exercise in AT (tendon: p=0.78; calf: p=0.76), while both increased (i.e., exercise-induced hypoalgesia) in controls (tendon: 9.5(17.8), p=0.03; calf: 21.3(22.7), p<0.01). Neither group experienced a post-exercise change in upper trapezius PPT (AT: p=0.35; control: p=0.37). There was no between-group difference in baseline calf (p=0.14) or tendon (p=0.19) PPT. However, baseline and post-exercise upper trapezius PPT were significantly higher in AT (baseline: 335.6(194.8); post-exercise: 321.2(170.1)) than in controls (baseline: 193.7(75.1), p<0.01; post-exercise: 198.1(79.1), p<0.01). DISCUSSION/SIGNIFICANCE OF FINDINGS: These findings suggest: (1) in persons with AT, central pain processing is altered at baseline, but unaffected in response to isometric fatiguing exercise; and (2) in persons with AT, peripheral pain processing is unaffected at baseline, but is altered in response to this mode and dosage of fatiguing isometric exercise.

